# Bax monomers form dimer units in the membrane that further self-assemble into multiple oligomeric species

**DOI:** 10.1038/ncomms9042

**Published:** 2015-08-14

**Authors:** Yamunadevi Subburaj, Katia Cosentino, Markus Axmann, Esteban Pedrueza-Villalmanzo, Eduard Hermann, Stephanie Bleicken, Joachim Spatz, Ana J. García-Sáez

**Affiliations:** 1 Membrane Biophysics, Max Planck Institute for Intelligent Systems, Heisenbergstrasse 3, 70569 Stuttgart, Germany; 2 Membrane Biophysics, Interfaculty Institute of Biochemistry, University of Tuebingen, Hoppe-Seyler-Strasse 4, 72076 Tuebingen, Germany; 3 New Materials and Biosystems, Max Planck Institute for Intelligent Systems, Heisenbergstrasse 3, 70569 Stuttgart, Germany

## Abstract

Bax is a key regulator of apoptosis that mediates the release of cytochrome c to the cytosol via oligomerization in the outer mitochondrial membrane before pore formation. However, the molecular mechanism of Bax assembly and regulation by other Bcl-2 members remains obscure. Here, by analysing the stoichiometry of Bax oligomers at the single-molecule level, we find that Bax binds to the membrane in a monomeric state and then self-assembles in <1 min. Strikingly, active Bax does not exist in a unique oligomeric state, but as several different species based on dimer units. Moreover, we show that cBid activates Bax without affecting its assembly, while Bcl-xL induces the dissociation of Bax oligomers. On the basis of our experimental data and theoretical modelling, we propose a new mechanism for the molecular pathway of Bax assembly to form the apoptotic pore.

## Abstract (web summary)

The proapoptotic protein Bax triggers cell death by forming pores in the outer mitochondrial membrane. Using single-particle TIRF imaging, the authors show that Bax binds the membrane in a monomeric state before forming dimers and multimers of dimers, which are disassembled by the survival protein Bcl-xL.

## Graphical abstract

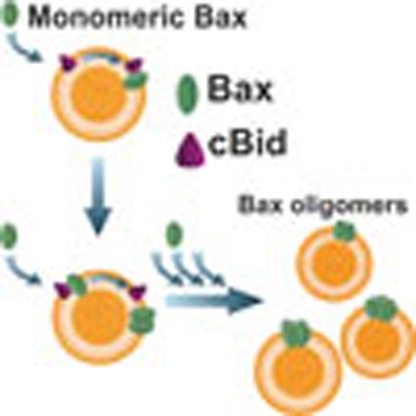

The proteins of the Bcl-2 family are key players in the mitochondrial pathway of apoptosis. They form a complex interaction network that determines the permeabilization of the mitochondrial outer membrane (MOM) and the release of cytochrome c, which is considered a point of no return in the cell suicide programme^1^. According to their putative role and the number of Bcl-2 homology (BH) domains they contain, the Bcl-2 proteins are further classified into three subgroups: (i) the antiapoptotic Bcl-2 proteins, such as Bcl-2, Mcl-1 and Bcl-xL, that contain all four BH domains and promote cell survival by inhibiting the proapoptotic family members; (ii) the executioner Bcl-2 proteins, including Bax and Bak, that contain domains BH1–3 and are believed to participate directly in MOM permeabilization; and (iii) the BH3-only proteins, such as Bid, PUMA or Noxa, that contain only the BH domain 3 and have evolved to sense diverse apoptotic stimuli and to initiate apoptosis by inducing Bax and Bak activation^2^.

Despite intense research, the molecular mechanism involved in MOM permeabilization in apoptosis remains one of the key questions in the field. During the last couple of decades, some features of Bax action have been uncovered. Under normal conditions, Bax is inactive as a monomer mostly located in the cytosol of healthy cells. In presence of apoptotic stimuli, Bax translocates to the MOM, where it undergoes a conformational change and oligomerizes to form the structures responsible for permeabilization of mitochondria^3^,^4^. Several models have been proposed to explain the regulation of Bax activity by other Bcl-2 family members, including the direct activation model^5^, the indirect activation model^6^, the unified model^7^ and the embedded together model^8^. Although some aspects remain controversial, the most spread view assumes that Bax activation and MOM permeabilization are induced by a subgroup of BH3-only proteins called direct activators, which includes Bim, PUMA and the cleaved form of Bid (cBid)^9^. Moreover, the activity of Bax can be inhibited by the prosurvival members of the Bcl-2 family via complex formation at the MOM and/or Bcl-xL-induced retrotranslocation of Bax to the cytosol^10^,^11^,^12^. Disruption of the complexes between Bax and the prosurvival Bcl-2 proteins mediated by the BH3-only proteins releases Bax, which can then induce MOM permeabilization^7^.

However, the nature of the structures formed by Bax in the membrane that induce MOM permeabilization remains obscure. On binding to the membrane, Bax alters its globular, mainly helical, cytosolic structure and adopts a different conformation that is extensively inserted in the lipid bilayer^13^,^14^,^15^. Recent crystallography studies with a truncated version of Bax in detergent media have identified a well-defined dimerization domain at the N-terminal half of the protein^16^. *In vitro* studies with model membranes have shown that Bax is able to form large and stable pores, of toroidal nature and tunable size^17^,^18^. Importantly, the oligomerization of Bax is an essential prerequisite for Bax-mediated MOM permeabilization^10^,^19^. Different oligomeric forms of Bax, ranging from dimers to high-molecular-weight clusters, have been detected in mitochondria using cross-linking and gel filtration^13^,^20^,^21^,^22^,^23^. However, none of these experimental approaches allows a precise estimation of the molecularity of membrane bound Bax. As a result, very little is known about the assembly pathway and oligomeric state of Bax during its activation and function in the lipid bilayer.

Here we have analysed the stoichiometry of individual Bax oligomers in the membrane over time by single-particle TIRF (total internal reflection fluorescence) microscopy^24^,^25^. We show that Bax molecules bind initially to the membrane as monomers, but associate quickly into dimers and higher-order oligomers. After equilibration, Bax exists in the membrane as different oligomeric species based on dimer units. We demonstrate that cBid activates Bax but does not affect its oligomerization pathway. In turn, Bcl-xL is able to inhibit Bax by dissociating previously activated Bax oligomers. On the basis of our experimental data and supported by mathematical modelling, we propose an unanticipated model for the assembly mechanism of Bax in the membrane.

## Results

### Activated Bax binds initially to the membrane as a monomer

**Figure 1:**
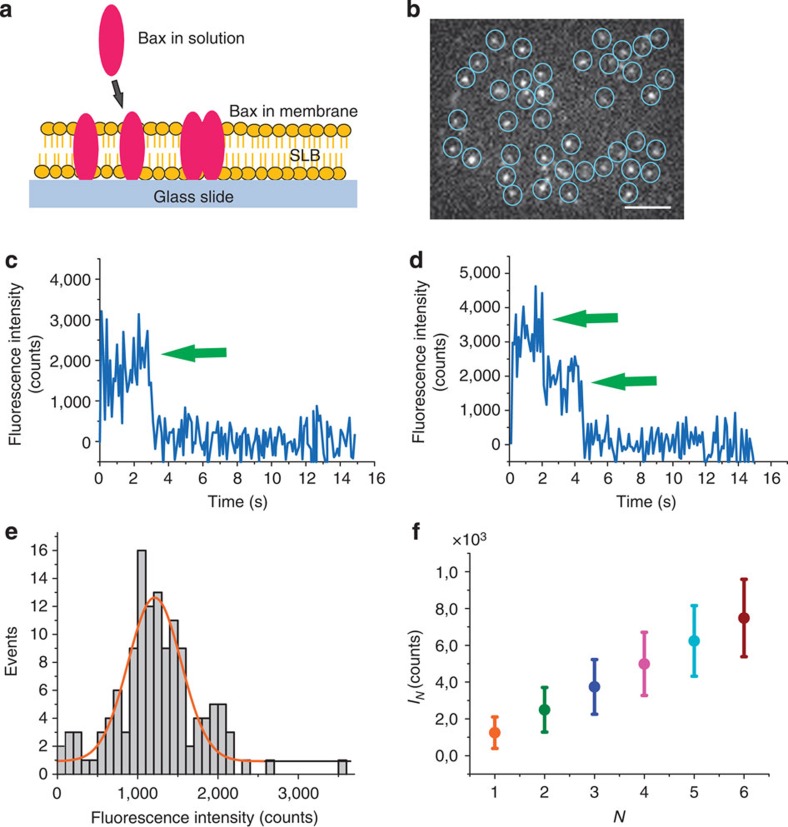
Detection and calibration of single BaxG fluorophores. (**a**) Schematic representation of the experimental setup showing a supported lipid bilayer with BaxG bound to it. (**b**) Epifluorescence image of BaxG molecules immobilized on a supported lipid bilayer. Scale bar, 1 μm. Each particle was detected and the fluorescence intensity measured over time. Detected particles are shown in circles to help visualization. (**c**,**d**) Fluorescence intensity of two representative individual particles showing photobleaching steps (arrow). (**e**) Histogram of the fluorescence intensity distribution of individual BaxG particles with a single bleaching event, fitted with a Gaussian to obtain the fluorescence intensity of a single fluorophore. (**f**) Mean fluorescence intensity (*μ*) and s.d. (*σ*) values calculated for individual particles containing one (orange), two (green), three (blue), four (magenta), five (cyan) and six (red) fluorophores.

To analyse the stoichiometry of single Bax oligomers in the membrane, we needed a chemically controlled system that avoids the unwanted effects due to the presence of multiple regulatory components and unknown factors, typical of a natural intracellular environment. In this sense, cell-free systems have been shown to recapitulate properly the key features of the Bcl-2 proteins observed during apoptosis^11^,^17^,^18^,^19^. Taking this into account, we implemented an *in vitro* reconstituted system based on recombinant, full-length fluorescently labelled Bax (BaxG)^17^,^18^ and supported lipid bilayers (SLBs) mimicking the composition of the MOM^26^. Using assays based on content release from lipid vesicles, we have shown previously that BaxG retains the pore-forming activity and regulation by other Bcl-2 proteins when compared with the wild-type protein^17^,^18^, indicating that fluorescence labelling does not alter the function of the protein. For our measurements, we used TIRF microscopy, which illuminates the sample with an evanescent wave immediately above the glass–buffer interface, therefore limiting the excitation to a thin volume of around 50–100 nm height, which comprises the planar membrane and the immediate solution above it. This increased the signal-to-noise ratio and allowed the detection of individual BaxG particles inserted into the SLBs (Fig. 1a,b).

We calibrated the fluorescence signal of the TIRF microscope before each experiment to avoid artifacts due to small changes in the optical setup (Fig. 1c–f). To this aim, we characterized the fluorescence intensity of individual BaxG monomers inserted in SLBs on a glass coverslip using photobleaching measurements. Samples were prepared by adding soluble, monomeric BaxG to the buffer above the SLB in the presence of unlabelled cBid and incubating for 1 h before imaging. Owing to the stochastic nature of the photobleaching process, the likelihood that two or more fluorophores in a particle will photobleach simultaneously is extremely low, typically leading to a stepwise decrease in the fluorescence intensity where the number of steps corresponds to the number of fluorophores in a particle. As fluorescent particles corresponding to BaxG monomers contain a single fluorophore, to calibrate their brightness we considered only particles that showed a unique photobleaching step (Fig. 1c,d). On the basis of the distribution of brightness of monomers, we plotted a histogram (Fig. 1e) from where we calculated the average monomer brightness and s.d. that was used to estimate the expected brightness of dimers up to hexamers (see Methods) (Fig. 1f).

**Figure 2:**
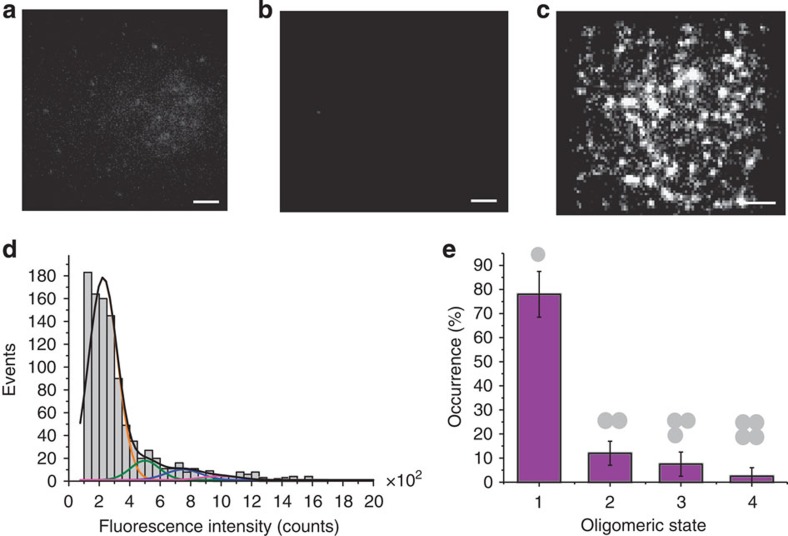
BaxG binds as a monomer to membranes containing CL in presence of cBid. Binding of BaxG particles to SLBs is negligible for membranes composed of phosphatidylcholine:cardiolipin (PC:CL) (8:2) in absence of cBid (**a**) and for membranes composed of pure PC in presence of cBid (**b**), but it is very efficient for membranes composed of PC:CL (8:2) in presence of cBid (**c**). The images show similar scaling to compare them visually. Scale bar, 1 μm. (**d**) Fluorescence intensity distribution of single BaxG particles added to SLBs made of PC:CL (8:2) in presence of cBid. Approximately 500 particles were analysed. The resulting histograms were fitted with a linear combination of four Gaussians to estimate the occurrence of particles containing one (orange), two (green), three (blue) and four (magenta) labelled molecules. The cumulative fit is shown in black. The area of the fitted Gaussians is proportional to the fraction of each species. (**e**) Percentage of BaxG monomers, dimers, trimers and tetramers calculated from the averaged distributions of species from three different experiments, after correction for partial labelling. The error bars correspond to the average error for each species.

We tested the specificity of the detection of BaxG particles by controlling the experimental conditions. We did not observe any protein bound to the SLBs in absence of cBid (Fig. 2a), where the weak signal detected corresponded to Bax molecules diffusing above the membrane without becoming inserted, or when the membrane was composed of pure phosphatidylcholine (Fig. 2b). In contrast, membrane binding of BaxG was very clear when the protein was incubated with unlabelled, wild-type cBid provided that the SLBs were composed of phosphatidylcholine:cardiolipin (8:2), a simple model of the MOM (Fig. 2c)^5^,^12^. Interestingly, BaxG particles did not diffuse in the membrane after insertion probably due to unspecific interactions with the solid substrate below the membrane. Under these conditions, we detected the isolated BaxG particles and measured their brightness, which was then plotted as a histogram to obtain the fluorescence intensity distribution (Fig. 2d). We fitted the histogram with a linear combination of Gaussian curves centred at the average intensities of BaxG monomers to tetramers^24^,^25^,^27^,^28^. The area under each curve was used to calculate the percentage of occurrence of each species, which was further corrected by taking into account that not all Bax molecules in a particle are labelled due to partial labelling (see Methods) (Fig. 2e). These results show that BaxG is mostly monomeric in the membrane under these experimental conditions and serve as a negative control for the oligomerization studies described below. The immobilization of the protein after membrane insertion hinders protein diffusion, which seems to prevent subsequent oligomerization. Importantly, these observations also indicate that on activation, Bax first binds to the membrane as a monomer.

### Membrane Bax exists as a mixture of species based on dimers

To allow the formation of Bax oligomers in the membrane that we can observe at the single-molecule level, we exploited a biochemical trick. We first incubated BaxG with large unilamellar vesicles (LUVs) made of phosphatidylcholine:cardiolipin (8:2) in presence of unlabelled cBid. We checked BaxG association to LUVs labelled with DiD using fluorescence cross-correlation spectroscopy. Interestingly, BaxG did not bind homogeneously to all liposomes in the sample, but only to a subset of them (Supplementary Fig. 1). In contrast to SLBs, LUVs contain free-standing bilayers and allow the free diffusion of Bax molecules bound to them. Therefore, in the LUV membranes the inserted Bax molecules should be able to oligomerize, if they have the tendency to do so. These results indicate that Bax binding to a vesicle promotes the binding of additional Bax molecules, in agreement with the ability of Bax to autoactivate^20^, and suggest that under our experimental conditions there is one complex per vesicle.

We then formed the SLBs using these BaxG-containing vesicles and imaged the single BaxG particles with TIRF microscopy as described above. The process of bilayer formation is shown in Supplementary Movies 1 and 2 and Supplementary Fig. 2. As expected, these particles did not diffuse, which we again attribute to the interaction with the glass support.

**Figure 3:**
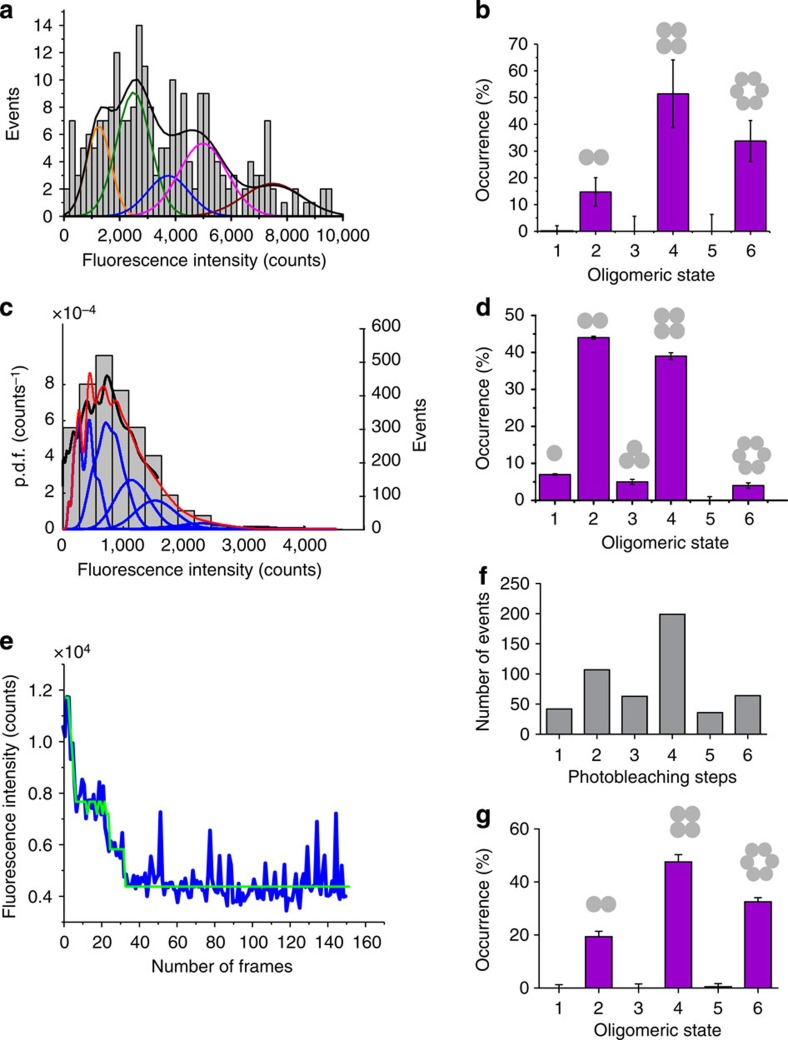
Bax exists in the membrane as a mixture of species based on dimer units. (**a**) Intensity distribution of individual BaxG particles bound to SLBs prepared from proteoliposomes after 1 h of incubation with BaxG and cBid. The resulting histogram was fitted with a linear combination of six Gaussians to estimate the occurrence of particles containing one (orange), two (green), three (blue), four (magenta) and six (red) labelled molecules. The cumulative fit is shown in black. (**b**) Percentage of occurrence of different oligomeric species calculated from the averaged distributions of species from three different experiments, after correction for partial labelling. The error bars correspond to the average error for each species. (**c**) Intensity distribution of a high number of individual BaxG particles (*n*=2,440) bound to SLBs prepared from proteoliposomes after 1 h of incubation with BaxG and cBid. The obtained brightness distribution was plotted as a probability density function (black line). The fit is shown in red and the *N*-mer contributions in blue. (**d**) Percentage of occurrence of different oligomeric species calculated from **c** after correction for partial labelling. (**e**) Representative photobleaching trace (blue) of a BaxG particle bound to SLBs prepared as in **a**. The green line shows the identified photobleaching steps after noise reduction. (**f**) Distribution of the number of photobleaching steps for 500 BaxG particles analysed as in **e**. (**g**) Percentage of occurrence of different BaxG oligomeric species calculated from **f** after correction for partial labelling.

Figure 3a shows a histogram of the distribution of fluorescence intensity for individual BaxG particles in SLBs created from LUVs incubated previously for 1 h with BaxG in presence of cBid. Direct visual inspection of the histogram shows a brightness distribution different from that of BaxG monomers shown in Fig. 2d, which suggests the existence of multiple high-order species. Figure 3b shows the distribution of Bax oligomers after correction for labelling efficiency (see Methods). Strikingly, not all possible oligomeric species were populated. We detected a mixture of dimers, tetramers and hexamers, where the levels corresponding to monomers, trimers or pentamers were much lower. There was a very small fraction of higher oligomeric species, probably octamers and/or decamers, as suggested by the presence of a small number of brighter particles (Fig. 3a). This population could not be included in the analysis due to intrinsic limitations of the technique to determine the stoichiometry of higher-order oligomers (see Methods), which does not challenge their existence. We took the immobilization of Bax particles on SLB formation to our advantage and analysed SLBs formed from LUVs incubated with BaxG and unlabelled cBid for different times between 1 min and 1 h. In these experiments, Bax molecules were allowed to oligomerize in the membrane of the LUVs for increasing amounts of time. Then, the SLBs were formed from these proteoliposomes, thus stopping BaxG diffusion and therefore the oligomerization process at the indicated times. We obtained very similar results (Supplementary Fig. 3) that indicate that Bax oligomerization is a relatively fast process and that the observed multiple oligomeric species corresponds to the equilibrium state of Bax in the membrane. In addition, the density of Bax particles detected on the SLBs increased with time, suggesting that membrane binding is the rate-limiting step in Bax oligomerization.

To control for the possibility of bias in the Gaussian fitting and of potential artifacts due to quenching effects in Bax oligomers, we analysed the stoichiometry of individual BaxG particles in the membrane by two alternative approaches, based on brightness analysis of a large number of particles with a probability density function (p.d.f.)^29^ and on single-molecule photobleaching counting^30^,^31^. In the p.d.f. analysis, we detected 2,440 particles in a single experiment and measured their brightness, which was used to calculate the corresponding p.d.f. (Fig. 3c). This was then fitted with a linear combination of the p.d.f. corresponding to the different *N*-mer species (see Methods), and corrected for partial labelling. Figure 3d shows the corresponding distribution of species, which is in agreement with the results of the Gaussian analysis.

In the photobleaching approach, time-lapse movies of the fluorescent particles are acquired. As photobleaching is a stochastic process, the probability that more than one fluorophore in one particle photobleaches simultaneously is very small. As a result, each fluorescent dye in a particle will stop emitting at a different time point. On the basis of this, fluorescence intensity traces with stepwise decreases in the signal due to the photobleaching of the individual fluorophores in a single particle were obtained. By counting the number of photobleaching steps detected with the NoRSE algorithm (see Methods) and correcting for partial labelling, the stoichiometry of the individual particles was calculated. Figure 3e shows a typical photobleaching trace for a single BaxG particle in SLBs prepared as explained above. By analysing about 500 particles, we obtained the histogram shown in Fig. 3f, which we corrected for partial labelling to calculate the stoichiometry of BaxG in the membrane (Fig. 3g). The results obtained also show several oligomeric species with enrichment in dimers, tetramers and hexamers, in perfect agreement with the brightness analysis (Fig. 3b,d). Moreover, analysis of the photobleaching step size in particles containing from one to six fluorophores demonstrated that Bax oligomerization does not affect the brightness of the individual fluorophores in a particle (Supplementary Fig. 4), which is important for a reliable brightness analysis.

### Effect of cBid and Bcl-xL on Bax oligomerization

Owing to the drastic consequences of Bax oligomerization and MOM permeabilization on cell fate, both the activation and activity of Bax are highly controlled. Although the most important regulators of Bax activity have been identified, little is known about the molecular mechanisms involved. To shed light onto this question, we studied the effect of an activator, cBid, and an inhibitor, Bcl-xL, on the assembly of Bax in the membrane.

**Figure 4:**
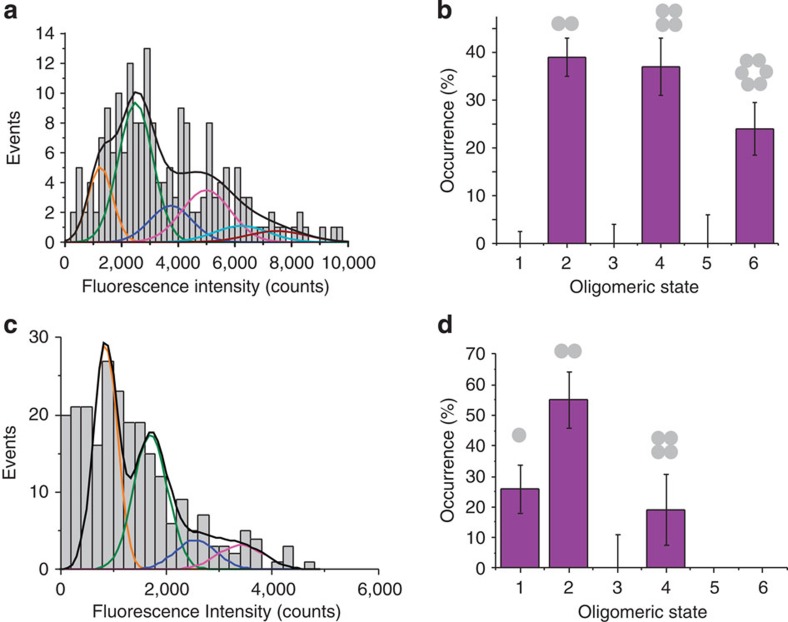
The extent of Bax oligomerization is not affected by cBid, while Bcl-xL induces the disassembly of Bax oligomers. (**a**) Intensity distribution of individual particles of heat-activated BaxG bound to the SLBs prepared from proteoliposomes after 1-h incubation at 40 °C. The resulting histogram was fitted with a linear combination of six Gaussians to estimate the occurrence of particles containing one (orange), two (green), three (blue), four (magenta), five (cyan) and six (red) labelled molecules. The cumulative fit is shown in black. Percentage of occurrence of different oligomeric species when BaxG is activated by heat (**b**) or when Bcl-xL is present in the membrane (**d**), calculated from the averaged distributions of species from three different experiments, after correction for partial labelling. The error bars correspond to the average error for each species. (**c**) Intensity distribution of single BaxG particles bound to SLBs prepared from proteoliposomes incubated for 1 h with BaxG and cBid, following addition of Bcl-xL and incubation during 1 h more. The colour code for Gaussian fitting is the same as in **a**.

Bid is inactive in the cytosol of healthy cells. In presence of apoptotic stimuli, it is cleaved to give the active form cBid, which then activates Bax via a yet unknown mechanism^32^,^33^. The ‘kiss-and-run' hypothesis assumes that cBid shortly interacts with Bax inducing an activating conformational change followed by dissociation of the two proteins^34^. However, up to now it remains unclear whether cBid has an additional role on the assembly pathway of Bax in the membrane. In the reconstituted system described here, BaxG remained inactive in solution in the absence of cBid (Fig. 2a). Addition of wild-type, unlabelled cBid induced membrane binding and oligomerization of BaxG (Fig. 2c and Fig. 3). To test the role of cBid on Bax oligomerization, we activated Bax via a cBid-independent mechanism. Mild heating at 42 °C has been shown to be sufficient to promote Bax oligomerization, pore activity and cytochrome c release from mitochondria^35^,^36^. We then analysed the stoichiometry of heat-activated BaxG in the lipid bilayer (Fig. 4a,b) and compared it with that of BaxG activated by unlabelled cBid (Fig. 3). As shown in Fig. 4b, heat-activated Bax also existed in the membrane as dimers, tetramers and hexamers, with a very similar distribution to that of Fig. 3. These results strongly suggest that cBid does not significantly affect the oligomerization mechanism of Bax in the membrane, although it is able to initiate it.

Several coexisting mechanisms have been proposed to explain how prosurvival Bcl-2 proteins inhibit Bax activity, including the sequestration of direct activators (like cBid)^7^, the retrotranslocation of Bax to the cytosol^10^, the competition for membrane binding and the formation of complexes in the membrane that inhibit Bax conformational change before oligomerization^11^. However, it is unknown whether Bcl-xL can disassemble active Bax oligomers in the membrane. To address this question, we incubated 2.5 nM BaxG with LUVs and 5 nM unlabelled cBid for 1 h and then added 2.5 nM full-length, unlabelled Bcl-xL and incubated the mix for one additional hour, followed by preparation of SLBs and image acquisition. The histogram in Fig. 4c shows a shift in the brightness distribution towards lower values of fluorescence intensity, suggesting a decrease in the oligomeric state of Bax particles promoted by Bcl-xL. Indeed, we found that Bcl-xL induced a distribution of BaxG species with a majority of dimers and a smaller percentage of monomers and tetramers. In contrast to Fig. 3, the hexamer population disappeared (Fig. 4d). Moreover, the average density of Bax molecules per area of SLB was decreased from 0.16±0.03 particles per μm^2^ to 0.11±0.01 particles per μm^2^ (five replicates) when the samples were incubated with Bcl-xL. These findings demonstrate that Bcl-xL is capable of disassembling activated Bax oligomers in the membrane, at least to a certain extent.

### Particle-based simulations of Bax self-assembly in membranes

The single-molecule analysis of Bax stoichiometry in the membrane described above shows that Bax binds to the membrane as a monomer, where it further oligomerizes to reach an equilibrium of species multiples of dimers. These findings suggest that species based on dimer units are more stable than oligomers with an uneven number of molecules, which has implications for the pathway of Bax self-assembly. To test if this assumption is compatible with the current knowledge of Bax activation by cBid, we performed spatial stochastic simulations. We used Smoldyn 2.32 (ref. 37) and simulated a 7 × 7 × 7-μm^3^ box containing 1,453 liposomes, 1,163 molecules of Bax and the double amount of molecules of cBid. This corresponded approximately to the experimental conditions of Bax membrane binding and oligomerization used here and the protein density of Bax in the SLBs. We included diffusion coefficients experimentally measured by FCS^12^,^18^ or reasonably estimated from them (see Supplementary Fig. 5). Our simulations started with all particles randomly distributed inside the box. We simulated the temporal evolution of such a system for 10 min using time steps of 50 μs. A scheme of the modelled mechanism and a list of the parameters used in the simulations are shown in Supplementary Fig. 5.

**Figure 5:**
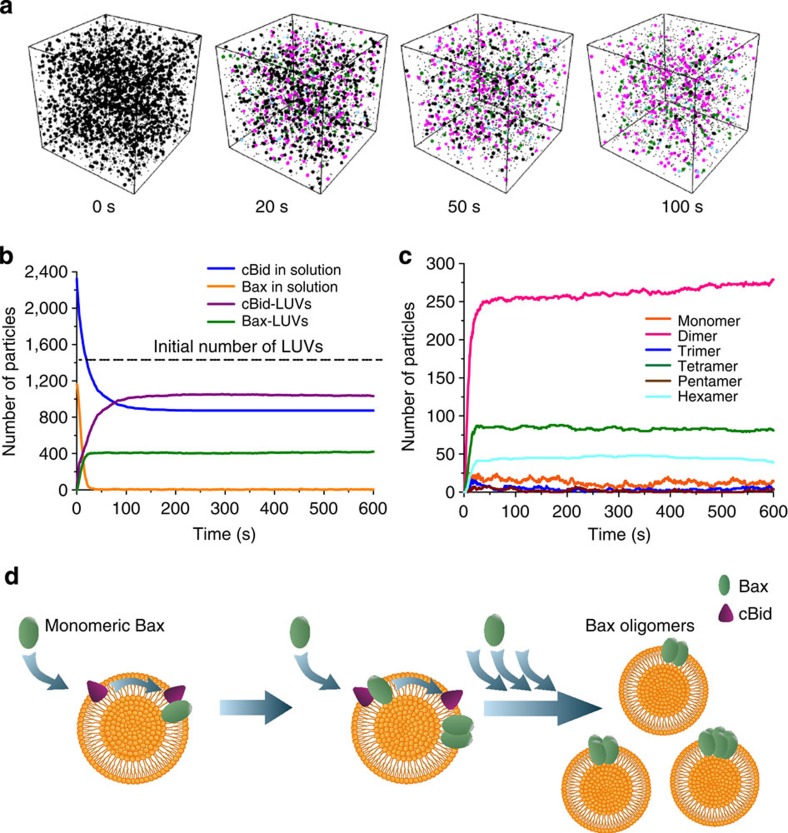
Particle-based simulation of Bax oligomerization on the surface of LUVs. Model for Bax self-assembly in the membrane. (**a**) Snapshots of the particle-based simulation at the indicated time points, showing the species presented in the system. The cubic box has a side length of 7 μm. Initially (0 s), 1,163 soluble Bax monomers, 2,326 cBid molecules and 1,453 LUV particles are uniformly distributed inside the box and are all represented as black spots. In the following time points, for clarity, only free LUVs (black), Bax dimers (magenta), Bax tetramers (green) and Bax hexamers (cyan) are represented as big particles. The other species are represented as small, black dots. (**b**) Time course of the simulated number of soluble Bax (yellow), soluble cBid (blue) and LUVs containing Bax (magenta) particles. The dashed line indicates the initial number of free LUVs. Only a subset of the LUVs in solution contains Bax molecules. (**c**) Time course of the simulated number of membrane-bound Bax species during 10 min. (**d**) Schematic view of the proposed autoactivation model for Bax oligomerization.

The core features of the experimental distribution of Bax oligomers could be satisfactorily reproduced with a simple model of oligomerization in the membrane. We assumed that Bax was recruited from solution by cBid or by other Bax molecules previously bound to LUVs (via autoactivation^38^). We also considered that Bax bound reversibly to the membrane as a monomer, unless forming a dimer with another Bax molecule stabilized it. Further addition of Bax molecules led to the formation of higher oligomers, where those with an even number of Bax molecules were favoured. Figure 5a–c shows the distribution of Bax oligomeric species at different time points obtained after running the simulation, which is enriched in oligomers with an even number of molecules similar to the experimental results. Importantly, this simple model also reproduced the binding of Bax to a subset of the liposomes (27% LUVs contained Bax), in agreement with the FCS experiments (Fig. 5b and Supplementary Fig. 1). By increasing the dissociation of monomeric Bax from LUVs and the relative stability of tetramers and hexamers, the simulation results could be better adjusted to the experimental distribution of species (Supplementary Fig. 5e,f). However, the biological implications of these tuned values should be taken with care due to the uncertainty of the experimental data. Finally, varying the concentration of cBid led to an increase in the total number of Bax particles oligomerizing in the vesicles (Supplementary Fig. 6a). When we tested experimentally this prediction of the model, the results obtained were in good agreement with the simulation (Supplementary Fig. 6b).

## Discussion

Here we have elucidated key aspects of the molecular mechanism of Bax assembly during its activation in the membrane. The use of reconstituted systems has been crucial to dissect the basic steps of Bax oligomerization with precise control of the presence of additional factors that regulate this process. We show that Bax molecules first insert into the membrane as monomers, which are quickly consumed in oligomerization reactions. This suggests that the structure of monomeric Bax embedded in the membrane is rather unstable with respect to its higher oligomeric forms. The immobilization of Bax monomers due to unspecific interactions with the solid support also implies that the oligomerization of Bax only starts after it is inserted via hydrophobic contacts in a protein conformation that spans the lipid bilayer. This is consistent with the conformational changes detected in Bax on activation^13^,^15^,^39^ and with kinetic studies that linked Bax insertion with oligomerization^19^. As a result, our findings strongly suggest that the initial step of Bax activation involves a conformational change that allows membrane insertion before dimer formation.

One of the most important findings in this study is that Bax exists in the membrane as multiple oligomeric species made of an even number of molecules. In our analysis, we could identify the presence of dimers, tetramers and hexamers. These results are supported by particle-based simulations, which could reproduce the experimental data with a relatively simple model that favoured particles with an even number of Bax molecules by increasing their probability of formation and decreasing their probability of dissociation. Interestingly, including autoactivation was necessary suggesting that this is a key property for Bax function. Our model considers an oligomerization mechanism compatible with the self-assembly of Bax on the LUV membrane as in our experiments. Additional association pathways, like oligomerization based on the condensation of dimer units diffusing in the membrane, that may play a role in the context of continuous membranes like the MOM, cannot be discarded. Taking this into account, we propose that Bax oligomers are based on dimer units, which are more stable than the complexes with uneven number of molecules.

Likely, there is also a small fraction of higher oligomeric species probably including octamers and decamers. However, under the single-molecule conditions of our study, where the concentration of Bax is the limiting factor, there is no significant amount of much bigger oligomers that would give rise to brighter particles. In this sense, the factor determining the endpoint of Bax oligomerization remains unresolved. One should nevertheless consider that the distribution of Bax oligomers might be affected by protein density in the membrane, which would allow the formation of higher oligomers at high Bax concentrations, also in the context of the MOM.

In this sense, our findings explain the variety of oligomeric forms found in previous studies^13^,^20^,^21^,^22^,^23^, which due to the bulk nature of the experiments or the strong dependence on the cross-linking efficiency could not directly associate the signal with specific oligomeric species. Moreover, these observations are in agreement with the toroidal model for Bax pore formation^17^,^40^. We have previously shown that Bax forms large, stable pores of tunable size^17^. Although from our data we cannot rule out which Bax species is responsible for membrane permeabilization, the ability of Bax to form multiple oligomeric species is in line with a flexible pore that would change size via accommodation of more or less dimers depending on protein concentration. Xu *et al.*^41^ have recently reported that even Bax monomers in presence of Bid BH3 peptides induced pores in lipid nanodiscs that could be detected by cryo-electron microscopy, strongly suggesting that all forms of Bax in the membrane are capable of inducing pores. On the basis of these reports and on the several different Bax oligomers described here, we propose that, in cells, Bax-induced MOM permeabilization would not be mediated by a unique, well-defined Bax pore structure, but rather by multiple Bax pores with different sizes and stoichiometries that adapt to Bax density at the MOM and add an additional level of regulation to Bax activity. This scenario would be consistent with previous data showing different release kinetics of cytochrome c and Smac from mitochondria in apoptotic cells^42^,^43^.

The idea of a dimeric organization unit is in good agreement with the stable dimerization domain presented by Czabotar *et al.*^16^ in the crystal structure for the N-terminal part of Bax in detergent, with the recent structural model for full-length, active Bax in the membrane^39^ and with additional low-resolution structural studies^44^,^45^,^46^. However, these structural approaches were so far unable to provide further insight into the nature of Bax oligomers. Moreover, the existence of multiple oligomeric forms of Bax in reversible equilibrium suggests that the protein/protein interactions involved in inter-dimer contacts are less strong than the intra-dimer ones. Indeed, the presence of multiple species is very likely an important reason why so far the structure of full-length active Bax in the membrane has remained elusive to high-resolution structural studies: there is not a unique conformation of Bax in the bulk lipid environment, but rather a mixture of structural arrangements that optimize inter-dimer and lipid contacts in the context of dimers, tetramers, hexamers, etc. In this scenario, our results are in excellent agreement with the large conformational flexibility we recently reported for the C-terminal part of the protein when it is embedded in the membrane^39^. This may have biological implications because, in cells, this flexibility in the interactions between dimers would also allow heteroligomerization with Bak, in agreement with previous observations^47^, as well as with other MOM components, as postulated in some models^48^.

We have also analysed the molecular effects of regulators of Bax activity on its oligomerization mechanism. We show that the assembly of Bax is comparable independently to the use of heat or cBid as activators, consistent with the ‘kiss-and-run' model for Bax activation^34^. These observations strongly suggest that the structural information for Bax self-assembly could be fully encoded in its own sequence and that the role of activators could merely be to induce the initial conformational change in Bax that enables membrane insertion and further self-association. This would mean that in the cellular context the extent of Bax oligomerization would also be independent of the activating factor, which is important, as several activators have been described *in vivo*^9^,^49^ and more may exist. Furthermore, as Bax-induced MOM permeabilization is a target for medical applications, our findings suggest that the design of small-molecule Bax activators affecting only the initial activation step should be enough to induce full Bax activity.

The functional importance of the multiple Bax oligomers is supported by our observations that the distribution of species can be modulated by Bax regulators. Bcl-xL was able to decrease the overall oligomerization state of Bax by displacing the distribution of species to mostly dimers and a small fraction of monomers and tetramers. As Bcl-xL was added 1 h after Bax activation by cBid, these results suggest a Bax/Bcl-xL interaction post Bax oligomerization and a new mechanism of Bcl-xL inhibition of Bax not reported so far. This scenario implies that the distribution of species is dynamic and depends on a set of reversible reactions that can be modulated by external factors. The effect of Bcl-xL can then be explained by two mechanisms, not mutually exclusive, that cannot be distinguished with our experimental approach. First, the appearance of Bax monomers suggests that Bcl-xL disrupts the Bax dimer units due to its higher affinity for membrane-inserted Bax monomers than the affinity between monomers themselves. As they are likely the basis for further oligomerization, dimer disassembly would additionally displace the equilibrium of the remaining Bax species towards smaller oligomers. These Bax/Bcl-xL complexes are likely to involve BH3 domain interactions, as reported in recent structural data by Ding and coworkers^50^. Second, it is also possible that Bcl-xL interrupts the interactions among dimer units in the oligomers. This would imply that Bcl-xL is able to invade Bax oligomers and disassemble them directly. In either case, this may have physiological implications for the regulation of Bax activity in cells and for the design of Bax inhibitors, as our data suggest that the most effective mechanism to block Bax action is the disruption of Bax dimers into monomers.

The experimental framework developed here opens new possibilities to study how Bcl-2 proteins interact with each other to regulate apoptosis. Taken together, our findings reveal an unanticipated core mechanism for Bax oligomerization and induction of MOM permeabilization in apoptosis (Fig. 5d). Supported by mathematical analysis, our results are consistent with a model in which the initial step of Bax activation would involve a conformational change that enables its insertion into the MOM as a monomer. Then, monomers would quickly self-assemble into higher-order oligomers, giving rise to a several different species based on multiples of dimers. This distribution of Bax species would exist under a reversible equilibrium that could be modulated by the membrane density of Bax as well as other additional factors, like Bcl-xL, and that would be responsible for the controlled permeabilization of the MOM.

## Methods

### Protein purification and labelling

Full-length mouse Bid and single-cysteine, full-length human Bax mutant (S4C, C62S and C126S), as well as full-length human Bcl-xL were expressed in *Escherichia coli* and purified^14^,^44^. Caspase-8 was used to cleave Bid (cBid). Protein quality was checked by SDS–polyacrylamide gel electrophoresis. Alexa488 (Invitrogen, Darmstadt, Germany) or Atto488 (Attotec, Siegen, Germany) dyes were covalently attached to the cysteine of the Bax mutant (BaxG) as described by the manufacturer. Excess of label was removed with desalting columns (BioRAD, Munich, Germany). The activity of the labelled proteins was controlled by giant unilamellar vesicle membrane permeabilization assay^36^. Labelling efficiency was calculated to be ∼70% or ∼80% by comparing protein and label concentrations with Bradford and spectrometer measurements.

### Supported lipid bilayers and large unilamellar vesicles

All lipids were purchased from Avanti Polar Lipids. For our experiments, lipid mixtures containing egg phosphatidylcholine and cardiolipin in an 8:2 ratio were used. The desired lipid mixtures were dissolved in chloroform. The solvent was evaporated under nitrogen flux and then subjected to vacuum for 1 h. The lipid mixtures were rehydrated to a final concentration of 10 mg ml^−1^ in 3 mM KCl, 1.5 mM KH_2_PO_4_, 8 mM Na_2_HPO_4_ and 150 mM NaCl, pH 7.2. A small aliquot of the multilamellar vesicle suspension (10 μl) was diluted in 140 μl of 150 mM NaCl, 10 mM Hepes, pH 7.4. The suspension was then vortexed and bath sonicated until obtaining small unilamellar vesicles. Glass slides used for preparation of bilayers were treated with piranha solution. The small unilamellar vesicle mixture was incubated at 37 °C for 2 min in the presence of 3 mM CaCl_2_ and was rinsed several times with 150 mM NaCl, 10 mM Hepes (pH7.4) to remove the non-fused vesicles^51^. The quality of the membrane as well as the absence of non-fused vesicles was controlled with confocal imaging and FRAP experiments. The prepared SLBs were incubated with 2.5 nM Bax and/or 5 nM cBid to be in the single-molecule regime.

To obtain proteoliposomes, LUVs were prepared as described in ref. 52. Lipids were dissolved in chloroform, and mixed in an appropriate ratio. The organic solvent was evaporated using a dry nitrogen stream and vacuum dried for at least 1 h. The dried lipid film was resuspended with 150 mM NaCl, 10 mM Hepes (pH7.4) to a final concentration of 1 mg ml^−1^. To prepare LUVs, the lipid solution was passed through five cycles of freezing and thawing after which they were manually extruded through a polycarbonate membrane of defined pore size (200 nm) using glass syringes.

LUVs were incubated with 2.5 nM Bax to be in the single-molecule regime, as well as 5 nM cBid and 2.5 nM Bcl-xL as indicated to form proteoliposomes. After the indicated incubation time, Bax-containing proteoliposomes were used to create SLBs without any lipid dilution (Supplementary Fig. 7).

### Microscopy

The glass slides (#1, 0.13–0.16-mm thickness, Menzel) used to prepare supported lipid bilayers were cleaned using a freshly prepared 3:1 mixture of concentrated sulfuric acid (95−97%, Baker) with hydrogen peroxide (30%, Merck) via immersion for at least 1 h. This cleaning procedure was specifically done to remove organic residues and to enhance the hydrophilicity of the glass surface. Slides were thoroughly rinsed with deionized water and sonicated with deionized water for 5 min. Before further usage, they were dried using a clean nitrogen stream. Individual slides were glued on the upper part of the measurement chamber (Lab-Tek 155411, Nunc, original borosilicate coverglass was manually removed) using glue. Chambers were used immediately for the preparation of supported lipid bilayers.

All experiments were performed using a microscopy system previously described^53^. Samples were excited on a modified Zeiss Axiovert 200M epifluorescence microscope at 37 °C using a diode laser (iBeam smart 488, Toptica, Germany) via a × 100, numerical aperture=1.46 Apochromat objective (Zeiss) for an illumination time *t*_ill_=1 ms and a delay of 90 ms between frames (number of frames=500) with an intensity of 5 kW cm^−^^2^ (measured at the sample).

### Fluorescence cross-correlation spectroscopy measurements

For fluorescence cross-correlation spectroscopy experiments, LUVs were labelled with 1,1-dioctadecyl-3,3,3,3-tetramethylindodicarbocyanine 4-chlorobenzenesulfonate salt (DiD-C18; Molecular Probes, Eugene, OR) at a molar concentration of 0.0003% and incubated with 20 nM BaxG and 100 nM cBid for 1 h. Experiments were performed on an LSM710 microscope with a C-Apochromat 40 × 1.2 water immersion objective (Zeiss, Oberkochen, Germany). Excitation light came from Ar-ion (488 nm) or HeNe lasers (633 nm). A spectral beam guide was used to separate emitted fluorescence. Autocorrelation and cross-correlation curves were computed and fitted as described in García-Sáez *et al.*^12^. The values were corrected for labelling effieciency of Bax.

### Stoichiometry analysis

The images acquired were used for the stoichiometry analysis based on the fluorescence intensity of the particles. For this purpose, individual particles with a single photobleaching step were detected via a threshold and their fluorescence intensity was estimated by fitting with a Gaussian. The estimated values were used to build a histogram of the distribution of fluorescence intensities that was fitted a Gaussian that provided the mean intensity *μ* and s.d. *σ* of a single fluorophore (Fig. 1e). With the estimated *μ* and *σ*, the fluorescence intensity of *N* co-localized fluorophores can be given by *μ*_*N*_*=Nμ*_1_±*N*^1/2^ *σ*_1_ (Fig. 1f). The number of Gaussians that can be fitted to the distribution of fluorescence intensity was estimated according to equation (1) (ref. 25):


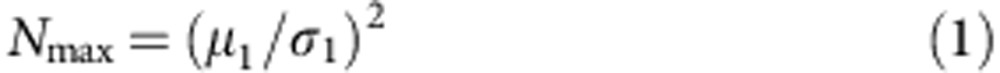


In our experimental data *N*_max_ is 6 and therefore we restricted our fittings up to hexamers. For determining the stoichiometry of Bax, ∼500 individual particles were detected and analysed for each experiment. The distribution of the fluorescence intensity of all the particles was fitted with a sum of six Gaussians using obtained *μ* and *σ* values. The Gaussian model used for the fit is given by equation (2):


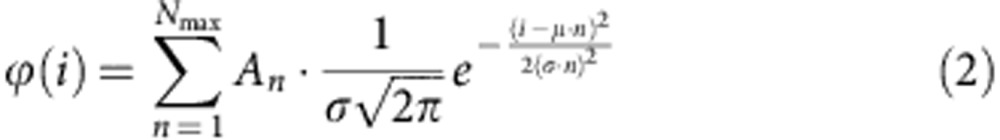


where *ϕ*(*i*) is the frequency of particles having intensity *i*, *n* is the component number and *A*_*n*_ is the area under the curve of component *n*. The area *A*_*n*_ under the curve of each component was used to estimate the percentage of occurrence of each species and at each time point as described in ref. 54. All the percentage values mentioned here were corrected considering that 80% of the protein is fluorescent (Supplementary Fig. 8). All experiments were done at least three times to ensure reproducibility of the results. The graphs with the distribution of species correspond to the average values obtained from the different experiments, and the error bars correspond to the averaged errors in the individual experiments, as they were larger than the s.d.

The single-molecule analysis by probability density function was done as described in Anderluh *et al.*^29^. Briefly, bright spots were localized by smoothing, non-maximum suppression and thresholding. The selected particles were fitted using a fast GPU-fitting routine^55^ to two-dimensional (2D) Gaussians using experimental parameters (pixel size) and for the background subtraction a maximum likelihood estimator for Poisson-distributed data. This algorithm provided the brightness value for each spot. These values were plotted as a p.d.f. Monomeric Bax particles were selected by choosing the image immediately before complete bleaching of the particle, as it will more likely be the brightness value of a single fluorophore. In this case, the probability of observing more than one single fluorophore is negligible. By convolution, the monomer brightness distribution was used to calculate the brightness distributions for higher oligomers. Finally, the overall brightness distribution was fitted by a linear combination of the *N*-mer contributions, similarly to the stoichiometry analysis by Gaussians.

Photobleaching analysis was done by using NoRSE algorithm^56^ that is based on Chung and Kennedy filter algorithm^57^. The data traces are imported and normalized by the maximum intensity. The forward and backward nonlinear filtering technique searches forward and backwards from each data point using selected window size to weigh the probability of occurrence of a sharp step. In our case, the size of the window is set to three or four frames depending on the data. The original noisy data is shown in blue (Fig. 3e) and the normalized data is shown in green. From the normalized data, the step sizes are calculated for each oligomeric species and are grouped according the step number.

### Particle-based simulations using Smoldyn

For the simulations we used Smoldyn Version 2.32 (ref. 37), which is a particle-based stochastic simulation tool for reaction–diffusion systems in user-defined geometries that is freely available at http://www.smoldyn.org/. This simulation framework has been successfully applied to biological systems^58^. Owing to its versatility regarding geometries and reactions on 2D surfaces embedded in a three-dimensional environment it is very well suited for the presented purpose. Smoldyn converts every reaction rate provided in the configuration script to either a probability (events per time step) for reactions of orders 0 and 1 or to a binding radius for reactions of order 2, which means that particles react once the distance between the two is smaller than the binding radius. In this framework, particles have no volume. Our simulation compartment is a 7 × 7 × 7-μm^3^ box. Initially, we randomly placed 1,163 Bax molecules, 1,453 LUV particles ((N° of LUV particles)=1.25 × (N° of Bax molecules)) and different amounts of cBid molecules into the box and let the system evolve over 10 min in time steps of 50 μs. We used diffusion coefficients and kinetic parameters as listed in Supplementary Fig. 5. The diffusion coefficients were measured experimentally^12^,^18^,^59^. For the kinetic parameters no experimental values are available, so we tested different combinations of parameters to reproduce our experimental findings as closely as possible. The Smoldyn script file for the simulation shown here is available on request from the corresponding author.

### Correction for partial labelling efficiency

The single-particle analysis provides the number of labelled monomers in each analysed particle. Since only a fraction of proteins is labelled (∼70–80%), this number does not necessarily correspond to the real stoichiometry of the particle, for example, a tetramer is most likely to have three labelled proteins and one unlabelled protein and thus appears as a trimer. To correct the distribution of oligomeric states for limited labelling efficiency, we considered that in a particle consisting of *N* monomers the number of labelled monomers, *m*, follows a binomial distribution (equation (3)):


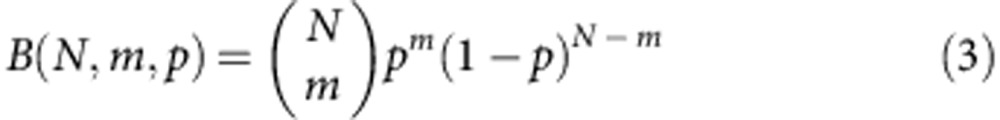


Here *B*(*N*, *m*, *p*) denotes the probability of having *m* labelled monomers in an *N*-mer with *p* being the fraction of labelled proteins in the sample. Keeping *p* fixed, *B* is a 2D matrix with entries >0 for *m*≤*N* and 0 else. Let *L*_*m*_ be the fraction of particles with *m* labelled monomers, as measured in the single-particle analysis, and *S*_*N*_ the fraction of particles consisting of *N* monomers (*N*-mers). Both quantities are connected via equation (4)


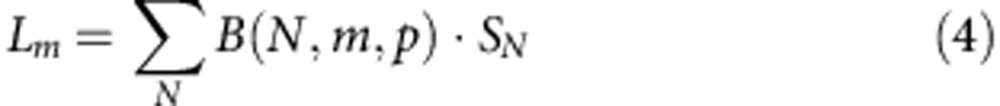


This yields a system of linear equations, which we solve for *S*_*N*_ to obtain the corrected distribution of oligomeric states using Matlab R2013b (The MathWorks Inc., Natick, MA). Since we do not include particles with more than six labelled monomers into our analysis, 1≤*m*≤6 and 1≤*N*≤6. Owing to experimental uncertainties, the measured *L*_*m*_ usually do not appear to originate from a perfect binomial distribution of labelled monomers. As a result, the solution for *S*_*N*_ may unexpectedly contain negative values, which indicates that the fraction of the corresponding *N*-mers is zero. Therefore, we set all *S*_*N*_<0 to zero and normalize the distribution such that 
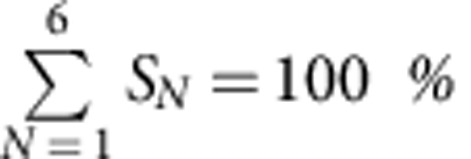
.

## Additional information

**How to cite this article:** Subburaj, Y. *et al.* Bax monomers form dimer units in the membrane that further self-assemble into multiple oligomeric species. *Nat. Commun.* 6:8042 doi: 10.1038/ncomms9042 (2015).

## Supplementary Material

Supplementary Information Supplementary Figures 1-8 and Supplementary References

Supplementary Movie 1 Formation of a supported lipid bilayer (red) by fusion of labelled large unilamellar vesicles (LUVs) previously incubated with BaxG. The movie starts a few seconds after addition of CaCl_2_. Total duration of the movie is 1 minute. For scale bar see Supplementary Figure 2.

Supplementary Movie 2 Appearance of single particles corresponding to labeled BaxG particles during the formation of a supported lipid bilayer by fusion of large unilamellar vesicles (LUVs). The movie starts a few seconds after addition of CaCl_2_. Total duration of the movie is 1 minute. For scale bar see Supplementary Figure 2.

Supplementary Movie 3 Photobleaching of Bax particles over time. Scale bar 5 μm.
