# Supplementary material for: Bax monomers form dimer units in the membrane that further self-assemble into multiple oligomeric species
Source: Nat Commun. 2015 Aug 14;6:8042. doi: 10.1038/ncomms9042 (PMC4557355; doi:10.1038/ncomms9042)
Supplement: Supplementary Information — Supplementary Figures 1-8 and Supplementary References [file ncomms9042-s1.pdf]

Supplementary Figures

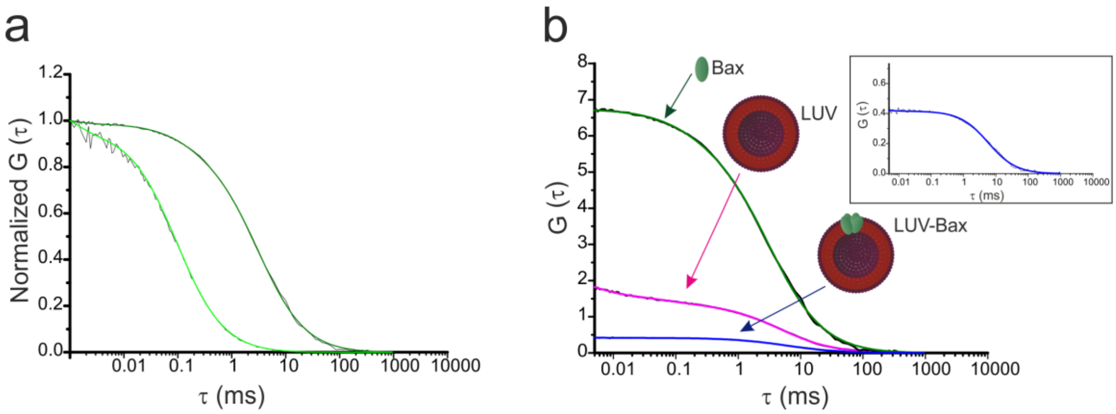

Supplementary Figure 1. Fluorescence correlation spectroscopy (FCS) analysis of BaxG binding to LUVs. a) Autocorrelation curves of BaxG alone in solution (light green) and BaxG incubated for 1h in a solution of LUVs in presence of cBid (dark green). The autocorrelation curves are normalized to *G* at time τ=0. The diffusion of BaxG presents a major slow component, indicating extensive binding to the vesicles. We also measured a decrease in the number of Bax particles in the detection volume, which is in agreement with the oligomerization of the protein in the LUVs.

b) Auto- and cross- correlation analysis of a suspension of LUVs (labeled with DiD) after 1h incubation with cBid-activated BaxG (20 nM). The fitted autocorrelation curves of BaxG and LUVs are shown in green and magenta, respectively. The amplitude of the cross-correlation curve (blue), with a cross correlation value of 28.13 ± 5.43 % (6 repetitions) with respect to the LUV particles, indicates that BaxG binds only to a small subset of the LUVs in the sample. The inset in the figure is an enlarged version of the CC-curve.

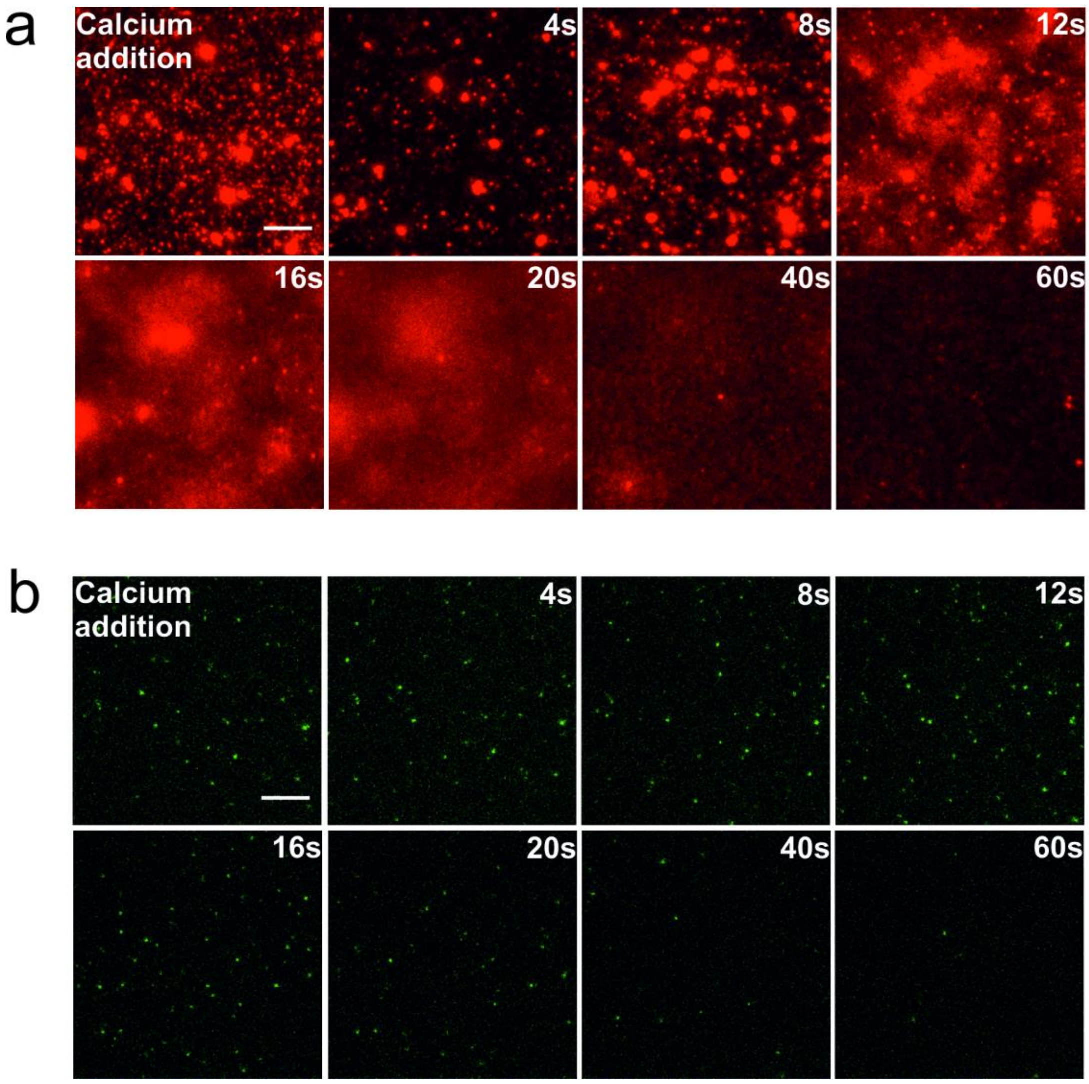

Supplementary Figure 2. Supported lipid bilayer formation (as shown in Supplementary movies 1 and 2). a) Formation of a supported lipid bilayer from the fusion of labeled large unilamellar vesicles (LUVs) (red). Image acquistion started a few seconds after addition of $CaCl_2$. Vesicles start to spread on the glass support and fuse with each other forming a uniform lipid bilayer where the dye can freely diffuse. b) Appearance of single particles (green) corresponding to labeled Bax complexes during the formation of the supported lipid bilayer. As only a small fraction of the LUVs contains Bax (see Supplementary Figure 1), the density of Bax particles in the supported bilayer is low and allows single particle detection. Due to some non-specific interactions with the glass support, Bax molecules cannot freely diffuse in the bilayer and finally undergo photobleaching. Scale bar is 5 μm.

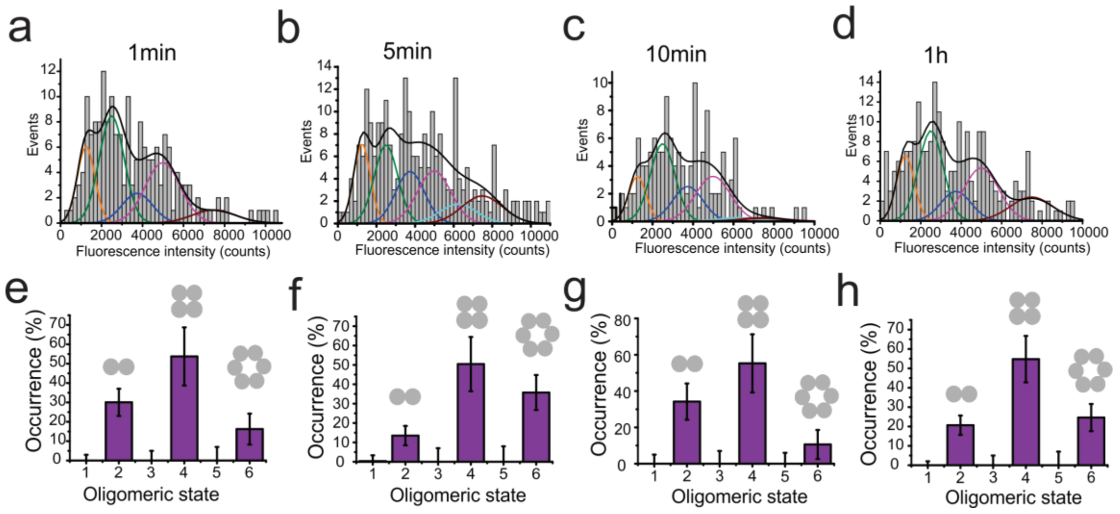

Supplementary Figure 3. Stoichiometry analysis of BaxG in SLBs prepared from proteoliposomes after different incubation times. a-d) Intensity distribution of single BaxG particles bound to SLBs prepared from proteoliposomes at incubation times: 1 min (a), 5 min (b), 10 min (c), 1 h (d) in presence of unlabeled cBid. The resulting histograms were fitted with a linear combination of six Gaussians in order to estimate the occurrence of particles containing one (orange), two (green), three (blue), four (magenta), five (cyan) and six (red) labeled molecules. The cumulative fit is shown in black. e-f) Percentage of occurrence of the different BaxG oligomeric species after different incubation times: 1 min (e), 5 min (f), 10 min (g), 1 h (h), calculated from (a-d) after correction for labeling efficiency.

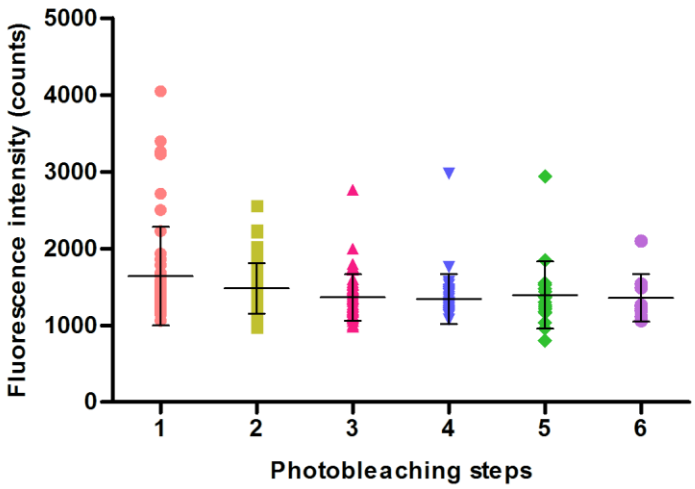

Supplementary Figure 4. Comparison of the fluorescence intensity of the photobleaching steps of Bax oligomers. We measured the change in fluorescence intensity of each step in the photobleaching traces corresponding to particles containing six, five, four, three, two and one fluorophore. There are no significant differences between the brightness of the fluorophores in the context of the oligomeric species analyzed, which discards potential quenching effects.

a

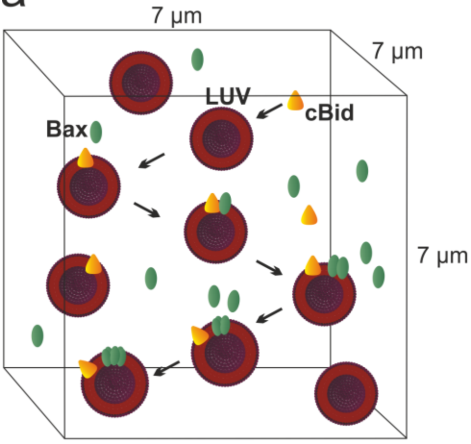

b

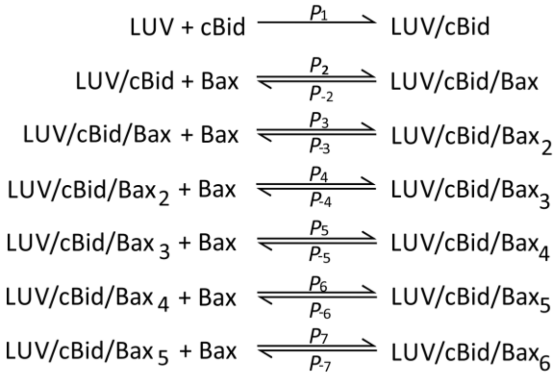

c

Parameter	Value (probability constant)	Value in Smoldyn (binding radius, µm)
P_1	0.01	0.00492522
P_2	0.05	0.0084198
P_{-2}	0.05	0 (U)
P_3	1	0.0228546
P_{-3}	0.0005	0 (U)
P_4	0.05	0.0084198
P_{-4}	0.05	0 (U)
P_5	1	0.0228546
P_{-5}	0.0005	0 (U)
P_6	0.05	0.0084198
P_{-6}	0.05	0 (U)
P_7	1	0.0228546
P_{-7}	0.0005	0 (U)

d

Particle	Diffusion coefficient ($\mu m^2 s^{-1}$)
Bax	60
cBid	80
LUV	1
LUV/cBid	1
LUV/cBid/Bax	1
LUV/cBid/Bax_2	1
LUV/cBid/Bax_3	1
LUV/cBid/Bax_4	1
LUV/cBid/Bax_5	1
LUV/cBid/Bax_6	1

e

Parameter	Value (probability constant)	Value in Smoldyn (binding radius, µm)
P_1	0.01	0.0228653
P_2	0.05	0.0390775
P_{-2}	0.2	0 (U)
P_3	1	0.106078
P_{-3}	0.01	0 (U)
P_4	0.05	0.0390775
P_{-4}	0.05	0 (U)
P_5	1	0.106078
P_{-5}	0.0005	0 (U)
P_6	0.05	0.0390775
P_{-6}	0.05	0 (U)
P_7	1	0.106078
P_{-7}	0.0025	0 (U)

f

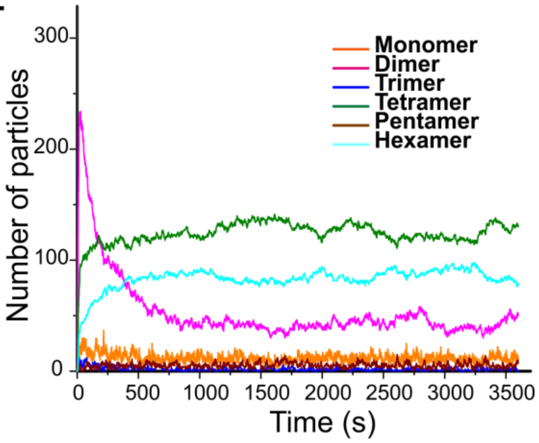

Supplementary Figure 5. Reactions and parameters used for the simulation of Bax binding to LUVs and oligomerization. a) Initially, 1163 Bax molecules (green), 2326 cBid molecules (yellow) and 1453 LUV particles (big purple spheres) are uniformly distributed inside a cubic box with a side length of 7 µm. All particles undergo Brownian diffusion and specific reactions. b) Scheme of all reactions in the simulation. The subscripts indicate the oligomeric state of Bax. c)

Kinetic parameters used in the simulation. The second column denotes the rate probability of the reactions (P_i) used in the script, and the third column indicates the binding radius computed by Smoldyn. Two particles react once the distance between the two is smaller than the binding radius. *(U)* denotes the unbinding radius. d) Diffusion coefficients used in the simulation. Diffusion coefficients of Bax and cBid in solution were estimated from the protein mass and using the formula $(D/D')^3 = m'/m$ [1]. As a reference, we used the measured diffusion coefficients of tBid and Bcl-xL[2]. Diffusion coefficient for LUVs was calculated from fluorescence correlation spectroscopy data. We assumed for all Bax oligomers-LUVs complexes the same diffusion coefficient. e) and f) Kinetic parameters have been tuned (e) to obtain a simulation of 1 hour (f) that adjusts to the experimental results better than the simpler model shown in Figure 5.

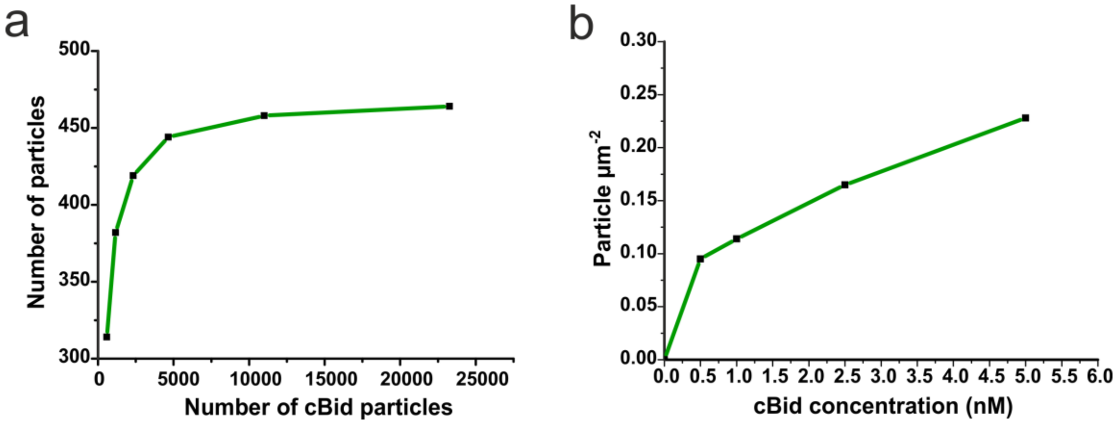

Supplementary Figure 6. Oligomerization of Bax at different concentrations of cBid. a) Simulated values of the number of Bax-oligomers formed in presence of different amounts of cBid molecules, obtained after 600s simulation according to the reaction scheme illustrated in figure S5b. b) Number of Bax-oligomers obtained experimentally at different cBid concentrations. LUVs were incubated with 2.5 nM Bax as well as 0.5, 1, 2.5 or 5 nM cBid to form proteoliposomes. After 1h incubation time, Bax-containing LUVs where used to create SLBs as described in the online methods. Although the conditions used are not exactly the same, qualitatively the trend observed is in good agreement with the simulations.

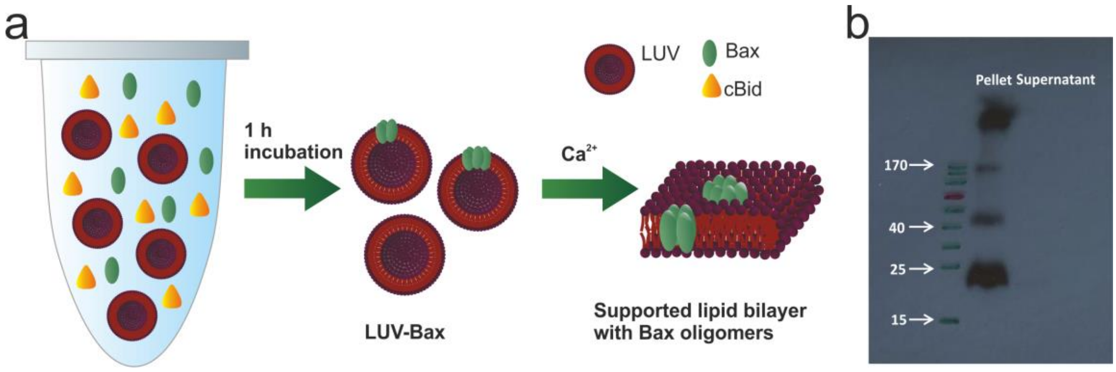

Supplementary Figure 7. Preparation of supported lipid bilayers containing Bax-G oligomers. a) Scheme of the protocol used for sample preparation. LUVs were incubated with 2.5 nM Bax and 5 nM cBid and incubated for 1h to form proteoliposomes. When indicated, the same sample was incubated with 2.5 nM Bcl-xL for an additional hour. After incubation, these liposomes were used to prepare planar bilayers. Unbound proteins and non-fused vesicles were removed by careful washing with buffer as described in the Methods section. b) Western blot of the supernatant (soluble fraction) and liposome pellet (containing membrane bound Bax) obtained from the ultracentrifugation of a sample where Bax-G was incubated with liposomes and cBid under the same conditions used in the single molecule experiments. The presence of Bax only in the pellet fraction demonstrates that all detectable protein is associated with the LUVs, and, therefore, there is no free Bax in solution that could stick to the glass surface.

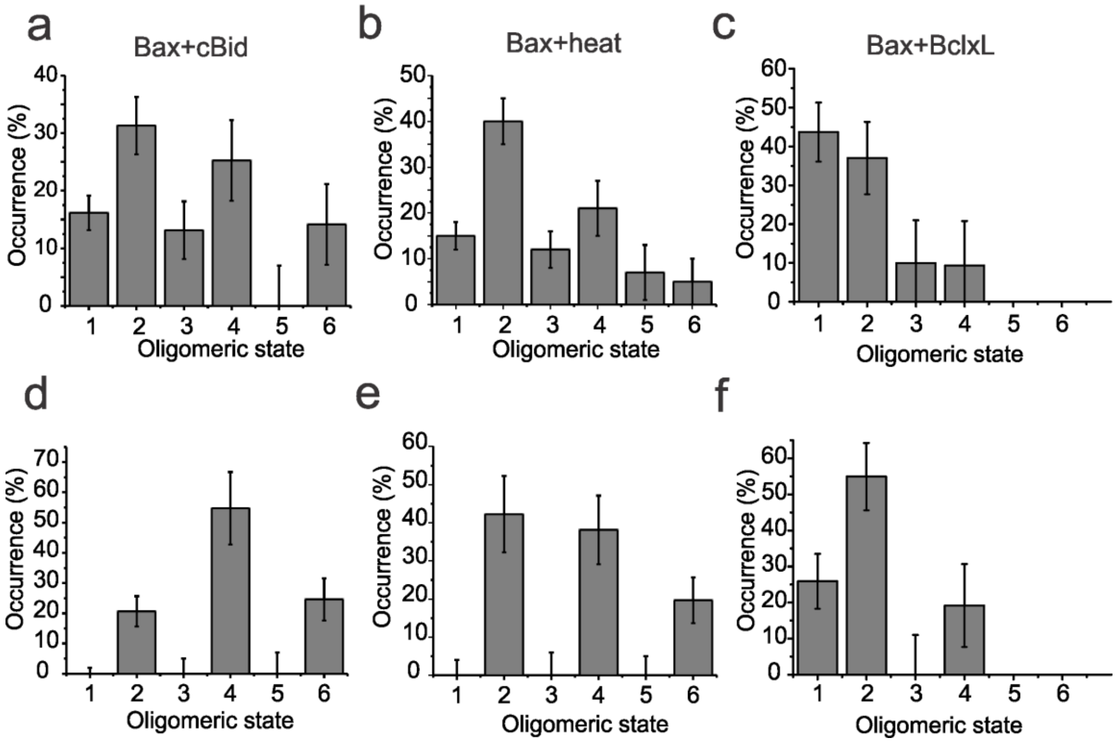

Supplementary Figure 8. Percentage of occurrence of different oligomeric species calculated before (a-c) and after (d-f) correction for partial labeling. a) and d) Percentage of occurrence calculated from the intensity distribution of individual BaxG particles bound to SLBs prepared from proteoliposomes after 1 h of incubation with BaxG and unlabeled cBid. b) and e) Percentage of occurrence calculated from the intensity distribution of individual particles of heat-activated BaxG bound to SLBs prepared from proteoliposomes after 1 h incubation at 40°C. c) and f) Percentage of occurrence calculated from the intensity distribution of individual BaxG particles bound to SLBs prepared from proteoliposomes incubated for 1h with BaxG and cBid, following addition of Bcl-xL and incubation during 1h more.

Supplementary References

1 Rehm, M. & Prehn, J. H. Systems modelling methodology for the analysis of apoptosis signal transduction and cell death decisions. *Methods* **61**, 165-173 (2013).

2 Garcia-Saez, A. J., Ries, J., Orzaez, M., Perez-Paya, E. & Schwille, P. Membrane promotes tBID interaction with BCL(XL). *Nat Struct Mol Biol* **16**, 1178-1185 (2009).
